# Trade-off Investment between Tonic Immobility and Mate Search in the Sweetpotato Weevil, *Cylas formicarius* (Coleoptera: Brentidae)

**DOI:** 10.3390/insects11110774

**Published:** 2020-11-09

**Authors:** Haoyong Ouyang, Pengxiang Wu, Runzhi Zhang, Muhammad Haseeb

**Affiliations:** 1 Center for Biological Control, College of Agriculture and Food Sciences, Florida A&M University, Tallahassee, FL 32307, USA; ouyanghaoyong@ioz.ac.cn; 2 State Key Laboratory of Integrated Management of Pest Insects and Rodents, Institute of Zoology, Chinese Academy of Sciences, Beijing 100101, China; wupengxiang@ioz.ac.cn; 3 College of Life Science, University of Chinese Academy of Sciences, No. 19(A) Yuquan Road, Shijingshan District, Beijing 100049, China

**Keywords:** prey, mate search, cost–benefit, anti-predator behavior, *Cylas formicarius*

## Abstract

**Simple Summary:**

Tonic immobility (TI) is considered as an important anti-predator strategy. In order to know the relationship between TI and mating behavior, we investigated the cost–benefit between TI and mate search in the sweetpotato weevil (SPW), *Cylas formicarius.* In this study, we used Y-tube olfactometer and determined that male mate searching increase after 3 h at night. Then, we confirmed that TI was affected by mate search in the males. Because the duration of TI of the male during mate search was significantly shorter duration than the males from the control. However, TI does not affect mate search in the females. Finally, the Y-tube olfactometer was used to compare the duration of mate search and the proportion of orientation towards the females in two artificial selection groups of the male SPWs. In this experiment, we found that mate search was affected by TI. Our study results provided a clear evidence that there is a trade-off between mate search and TI behavior in males of the SPW. Further, we quantified investment of anti-predator behavior and mating behavior by measuring the duration of TI and mate search in the SPW. We believe, these methods can be useful to determine the cost–benefit between anti-predator behaviors in the prey(s) and predator(s) in other species too.

**Abstract:**

Tonic immobility (TI) is a well-known anti-predator strategy adopted by diverse preys. Numerous studies on the cost–benefit involve in TI have been reported. Although, some studies have reported the effect of mating behavior on TI, few studies highlight the phases of mate search. In the present study, we investigated the relationship between mate search and TI behavior in the sweetpotato weevil (SPW), *Cylas formicarius* (Coleoptera: Brentidae). First, we found the most active mate search period of male SPW within 24 h. Then, we measured whether the duration of TI of virgin male and female were affected during the mate search. In the end, the Y-tube olfactometer was used to compare the duration of mate search and the proportion of orientation towards the females in two artificial selection groups of the male SPW with longer and shorter duration of TI. Our study confirmed that male mate searching increase after 3 h at night, and up to 73% at midnight, TI was affected by mate search in male, because the duration of TI of the male during mate search (Mean ± SE = 214.53 ± 22.74 s) was significantly shorter duration than the control (679.64 ± 69.77 s). However, mate search did not affect the strength of TI in the females tested. This study determined that mate search was affected by TI due to males from the group with shorter duration of TI who had 28% higher proportion of orientation towards the females than the males with longer duration of TI. Investment trade-off between TI and mate search was confirmed in the males of the SPW.

## 1. Introduction

Apparent death, colloquially known as playing dead, or feigning death is a behavior in which animals take on the appearance of being dead. This form of animal deception is an adaptive behavior also known as tonic immobility (TI) or thanatosis.

When prey is stimulated by predator or environment, the body becomes motionless and no longer responds to external stimulation. TI has been found in a wide range of taxa [1]. In the invertebrates, spiders [2], butterflies [3], beetle [4,5,6], juvenile dragonflies [7], and ants [8] were confirmed for the TI. In vertebrates, the TI of birds [9,10], fishes [11], amphibians [12], and snake [13,14] were reported earlier. Among them, the TI activity of arthropod has received considerable attention. Animals always face the threat of predator(s) which effect their feeding, breeding, and mortality. In order to reduce the influence of predation, prey evolves a wide variety of morphological, physiological, and behavioral traits [15]. The interaction between predator and prey can be described in seven stages, two individuals being in proximity, detection, identification, reduce separation, contacting, subduing, and consuming [16,17]. TI is considered as an anti-predator behavior to minimize the damage from predator base in the stages of subduing and consuming. Though the function of TI in arthropods is still unclear and there is increasing evidence that suggests TI plays a critical role in anti-predator behavior.

Even though TI always been considered an anti-predator behavior, there are also some costs affiliated to it. A previous study compared the duration of TI of *T. castaneum* in five different populations, the population which were maintained in the lab without predators for at least 100 generation showed shortest duration of TI. This suggested that TI is costly in an environment without predators [18]. Fleeing and aggressively attacking are considered as anti-predator behavior to reduce damage base in the various stages of predator subduing and consumption. Ants, birds, mites are the major predator of the sweetpotato weevil (SPW) in nature [6]. As the previous study reported, it is difficult to quantify the investment of anti-predator behavior [19]. However, we can measure the duration of TI to quantify anti-predator behavior. So, an animal with TI could be a suitable model to figure out the trade-off investment between anti-predator behavior and other behavior [20]. For trade-off, one characteristic cannot increase without a decrease in another. Because an organism has finite resource (energy, cost, space, time, food, etc.) [21].

Sweetpotato weevil (SPW), *Cylas formicarius* (Coleoptera: Brentidae), exhibits TI after stimulation [22]. When SPW is stimulated by a predator or other external stimuli, the body becomes motionless, and fall from the host plants. The predators of the SPW, including mite, ants, and mice have been reported [22,23]. As a result, the predator finds it hard to locate the position of the SPW; TI is considered as an effective defensive strategy that has evolved in *C. formicarius* to reduce the damage from predators. It is a good model to study TI because several studies of TI of the SPW have been reported [19,24,25].

Mating is a crucial behavior for animal reproduction, but it always requires many investing resources. Previous studies confirmed that there is trade-off between reproduction and other costly traits, such as dispersal capability [26] and immune performance [27]. Mating behavior can be divided into several phases, including mate searching, copulation, insemination, and post-copulatory interaction [28]. A previous study demonstrated that the duration of TI of adult female SPW was decreased after copulation, but the mate search does not affect the strength of TI [20]. Mate search is considered as an energetically costly behavior [29,30]. At the same time, it increases male and female mortality during mate search [31,32,33]. For example, male golden orb-web spider survival rate is extremely low (36%) during the period of mate search [34]. In the mate search behavior of the SPW, females using volatile sex pheromone to attracts males in a fixed position. At the same time, the male keeps walking to look for females [35].

TI has frequently been investigated in physiological mechanisms, function, adaptive significance, and cost–benefit analyses [36,37,38,39]. However, few studies analyze the cost–benefit TI and mate search. Cost–benefit of TI and mating behavior can contribute towards finding out the mechanism of TI. Kuriwada used the adhesive plaster to prevent them copulation and confirmed that there is no significant effect of encounter with opposite sex to TI [19]. However, we cannot define this phase as a mate search. The SPW with adhesive plaster still can touch each other. In the present study, we examined the following points to work out the trade-off between mate searching and TI behavior. First, to determine the most active mate search period of males of the SPW within 24 h. Second, to investigate whether the duration of TI of virgin males and females were affected during mate search. In a Y-tube olfactometer study, we compared the duration of mate search and orientation in two artificially selected groups. We hypothesized that starting night to midnight times are the most active mate search periods for the sweetpotato weevil, because a previous study found that the SPW prefer to copulate at night [40]. Mate searching behavior would reduce the duration of TI in males and females. At the same time, the virgin male with longer duration would show a longer period of mate search, because of a trade-off investment between anti-predator and reproduction behavior.

## 2. Materials and Methods

### 2.1. Insects Culture

Mass-reared virgin adult males and females of the SWD were used in this study. Original SPW colony was obtained from the Tropical Research and Education Center, University of Florida, Homestead, Florida, USA, in June 2018. The SPW were reared on sweetpotato tubers under the following conditions: 25 ± 1 °C, 70–90% relative humidity and a photoperiod of 12:12 h (light on: 06:00–17:59). The SPW were reared for eight generations at the Center of Biological control, Florida A&M University, Tallahassee, FL, USA. Adult males and females of the SPW were distinguished from the distal segments of antenna. Adults come out from the infested tubers in 30–40 days after inoculation [41]. Newly emerged males and females were maintained in separate plastic dishes. Each dish contained 30 adult weevils and 50 g sweetpotato tubers.

### 2.2. Observations of TI Behavior

In order to prevent weevil from same-sex sexual behavior and disturbance, individual male and female were kept in separate plastic containers with 5 g sweetpotato tubers for 24 h before the observation of TI performance [6]. A 14–16 days old adult of the SPW was collected in a plastic dish to observe the duration of TI. Each weevil was weighted by the electronic balance (A&D ER-60A, Tokyo, Japan) before it was isolated in a separate container. Each weevil was induced TI by using a forceps (Bio Quip 4750, Rancho Dominguez, CA, USA) to grasp the abdomen and then dropping it into its a dish from a height of about 2 cm, at the same time, the digital video camera (Canon M50 with EF-M 28/3.5 lens, Tokyo, Japan) was used to record the duration of TI behavior for 40 min. We defined the period between the dropping of a weevil into the dish and the detection of first visible movements of legs or antenna as the duration of TI. If the weevil was failed to respond to the first stimulation, we repeated it to two more times. In the end, if the weevil failed to response all three artificial stimulation, the duration of TI was recorded 0.1 s. We used the Gamma distribution and log link function in GLMMs, the duration of TI was not used at 0 level.

### 2.3. Artificial Selection

TI is considered as a heritable trait and suffered from natural selection [42,43]. Because the individual of the SPW showed a differential expression of TI, we used the two-way artificial selection base in the duration of TI [42]. The group of the SPW with longer duration of TI was defined as L-strain, the other group with shorter duration and lower frequency of TI was defined as S-strain. Fifty males and fifty females of the SPW were randomly selected from the original culture and measured their duration of TI. Ten of each male and female with the shortest duration of TI were selected to S-strain as F0, at the same time, ten of each male and female with the longest duration of TI were selected to L-strain as F0. Both males and females from F0 strain were introduced in a plastic container with the sweetpotato tubers for feeding, mating, and oviposition for a week. From the F1 generation of adult SPW eclosion, we randomly collected 50 sexually mature males and 50 mature females from each strain. Then, we measured their duration of TI. Ten of each female and ten of each male with the shortest duration of TI in S-strain were selected as S-1. Similarly, ten females and ten males with the longest duration of TI in L-strain were selected as L-1. Finally, the weevils from S-1 and L-1 were respectively introduced in a container to reproduce F2 generation. We used the same procedure to continue for eight generations for each strain. Each experiment was replicated five times. In the F7 generation, we measured the duration of TI of male and female weevils from L and S strain. Both male (mean ± SE = 1205.50 ± 70.29 s, *n* = 50) and female (1002.73 ± 56.44 s, *n* = 51) weevils from L-strain showed significant longer duration of TI than male (mean = 234.25 ± 20.8 s, *n* = 50) and female (171.37 ± 10.85 s, *n* = 52) weevils from S-strain.

### 2.4. Y-Tube Olfactometer

**Figure 1:**
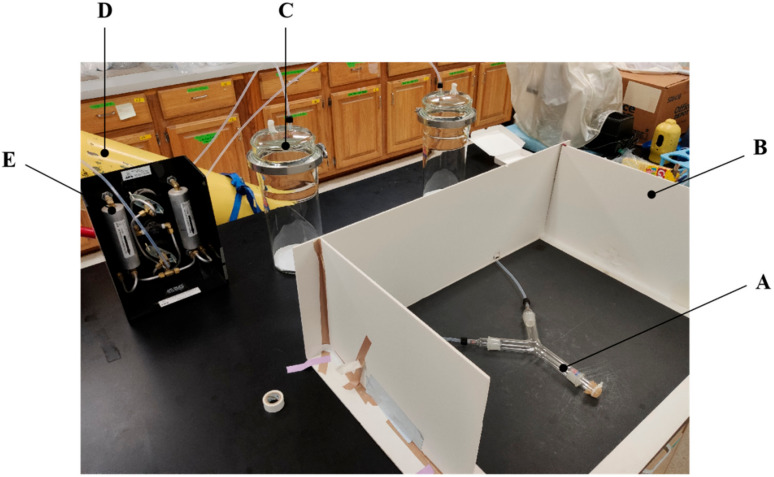
The Y-tube olfactometer for observing the mate search behavior in the sweetpotato weevil (SPW). (A) Y-shaped glass tube; (B) The wall used to prevent male SPW from being influenced by external factors; (C) Odor sources jar; (D) Clean air gasholder; (E) Anemometer.

For the observation and measurement of the adult male SPW mate search duration and the orientation to female stimuli, we used the glass Y-tube olfactometer which allowed observing and recording of walking insects behavior influenced by the two odors. The Y-tube olfactometer including a Y-shaped glass tube and two arms whose inner diameter was 3.5 cm, each arm was 15 cm long, and the angle between each arm and the main body was 45°. The end of each arm was connected with a glass jar (10 cm in diameter, 30 cm in height) which was used to place the odor source. In order to keep clean and constant air in all experiments at 300 mL/min, the glass jar was joint with clean air gasholder and anemometer. The outlet tubes were covered with gauze to prevent the insects in the entrance of Y-tube olfactometer (as Figure 1). The choice line was defined as the position at 5 cm from the junction.

### 2.5. Experiment 1: Ability of Searching Mates in Different Period

The adult virgin males (14–16 days) of the SPW that had been kept in isolation for a day and weighted by electronic balance were used in this experiment. In order to observe the SPW mate searching behavior for a day, we used the Y-tube olfactometer to record the orientation of males towards the female and the control (without insects). The experiment was conducted in eight different period, 06:00–08:59 h, 09:00–11:59 h, 12:09–14:59 h, 15:00–17:59 h, 18:00-20:59 h, 21:09–23:59 h, 00:00–02:59 h, and 03:00–05:59 h. During the period of 06:00–17:59 h, trails were conducted under light. In the other 12 h, trails were carried out under dark conditions. We observed the SPW by red light under dark condition. At the beginning of the trail, three virgin female adult SPW were introduced to one of the glass jars as odor source. Then, we turned on the fresh air gasholder and adjusted anemometer to kept constant clean air in all experiments at 300 mL/min. After 10 min, the individual one virgin male adult of the SPW was introduced into the base of the entrance. If the male cross choice line for 2 min, we record the orientation. However, if it fails to cross the choice line and stay for 2 min within 25 min, we do not record it. To avoid any possible asymmetries in the experimental set-up, including environmental factors, location effects, olfactometer, and jars were cleaned using ethyl alcohol (95%). The trial of each period was replicated 15 times. Two jars were switched after 5 replications.

### 2.6. Experiment 2: Effects on TI

For the purpose of distinguishing mate searching from a different phase of mating behavior, the trial started from 21:00 to 3:00 h. Because experiment 1 showed that more males of the SPW oriented toward the females from 21:00 to 3:00 h. In this trial, we compare the duration of TI between mate searching males of the SPW and resting males (control). The 14–16 days old adult virgin males and females (kept in isolation for a day) and weighted by an electronic balance were used in this experiment. After the isolation of male and female SPW, a virgin male was transferred into a Petri dish (15 cm diameter × 2 cm height) containing 1 g of sweetpotato tuber. Then, a female and 1 g sweetpotato tuber were introduced into another smaller Petri dish (5 cm diameter × 2 cm height) which was covered with gauze. Then, the smaller dish was transferred to the big dish. During the trial, the gauze was used to prevent the male from mounting and touching female with their antennae. However, the male still could search the female normally. After 30 min, the duration of TI of the male SPW that was crawling on the gauze was measured (mate searching group). The smaller Petri dish with 1 g sweetpotato root and gauze was placed into the large Petri dish which containing a virgin male and 1 g sweetpotato root, after 30 min the duration of TI of male SPW was measured (control group). Each group was replicated 30 times.

**Figure 2:**
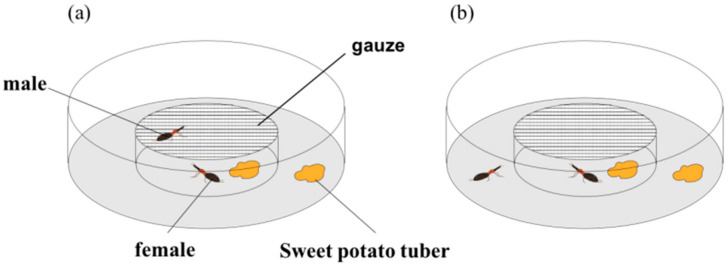
Arena of male searching for a mate: Phases of mate search of male of the SPW: Gauze was used to prevent interaction between male and female. (**a**) After the start of the experiment 30 min if males climb on gauze, it was defined as mate search. TI was measured for each male, (**b**) Orientation of male failed response to female.

To figure out whether the males will affect the duration of TI of females, we used the same method as mentioned above to measure the duration of TI in the females. A virgin female was transferred into a Petri dish (15 cm diameter × 2 cm height) containing 1 g of sweetpotato root, and then a smaller Petri dish (5 cm diameter × 2 cm height) with a male, 1 g of sweetpotato root and gauze was placed into the large Petri dish with a female. After 30 min, the duration of TI of female SPW was measured (mate searching group). The smaller Petri dish with 1 g sweetpotato root and gauze was introduced into a small Petri dish containing a virgin female and 1 g sweetpotato root (Figure 2). After 30 min, we measured the duration of TI of female SPW (control group). Each group was replicated 30 times. We observed the SPW by red light under dark condition.

### 2.7. Experiment 3: Effects on Mate Searching

In order to determine if males with different heritable TI traits have different mate searching performance innately. Three virgin females (14–16 days old) were randomly chosen from the mass-reared weevils. The 14–16 males were chosen from the L-8 and S-8 strain. One day before testing, the males and females were isolated for 24 h and weighed by the electronic balance. During this experiment, we used a Y-tube olfactometer to record the duration of mate search and orientation in L-8 and S-8 males. Three females were introduced into one of the jars as odor source. Then, we turned on the fresh air gasholder and adjusted anemometer to kept constant clean air in all experiments at 0.3 mL/min. After 10 min, the individual virgin male from L-8 strain was introduced into the entrance of the Y-tube olfactometer. If the male SPW induced TI, the duration of TI was recorded as *T_TI_* However, when the weevil fails to respond to the first stimulation, we repeated it two more times. Finally, if the weevil fails to respond to all three artificial stimulation, the duration of TI was recorded as 0.1 s. The same reason as measurement of duration of TI in SPW. The duration of mate search (*T_MS_*) of the individual was specified as the period between the detection of first visible movement of the SPW and it had made a decision (same judgment method using the choice line in the experiment 1). So *T_MS_* =*T* (totally observation time) − *T_TI_*. At the same time, we used the same method as experiment 1 to record orientation. The same procedure was conducted to measure the duration of mate search and orientation in S-strain. We observed the SPW using red light under dark conditions.

### 2.8. Statistical Analysis

All data were analyzed with R 3.4.3 [44], and graphs plotted using “ggplot” {ggplot2} [45]. We used generalized linear mixed models (GLMMs) to analyze all data by lmer function in the lm4 package [46]. To compare the orientation of male in different period, we used Gamma distribution and log link function. Time was fix factor, the age, weight, and replication were the random factor. To compare the duration of TI of the SPW in two phases of the SPW, we used Gamma distribution and log link function. The phase of mate of SPW was fix factor, the age, weight, and replication were the random factor. To confirm the effect of TI to mate search, we used binomial distribution and logit link function. The strain was fix factor, the age, weight, and replication were the random factor (where 1 = successfully orient to female 0 = fail to find the female). Data were analyzed with binomial GLMM with logit link. After each model was fitted, the significance of different time, phase and strain of the SPW were assessed by a likelihood ratio test between models, with and without the factor of interest, using *C^2^* testing in “drop1{stats}” [44,47].

## 3. Results

### 3.1. Experiment 1: Ability of Searching Mate

The orientation rate of male to female in eight different period is shown in Table 1. The male became more active in the period of 21:00 h. The orientation rate of males to females increased significantly in period of 21:00–23:59 h. The orientation rate of male to females further increased rapidly in the period of 00:00–02:59 h, reaching a peak (73.3 ± 11.4%). After this peak, males SPW were no longer attracted to the females. Consequently, the probability of orientating towards females was decreased. Finally, the orientation rate of male to female become stable during the 6:00–20:59 h.

### 3.2. Experiment 2: Effects on TI

**Figure 3:**
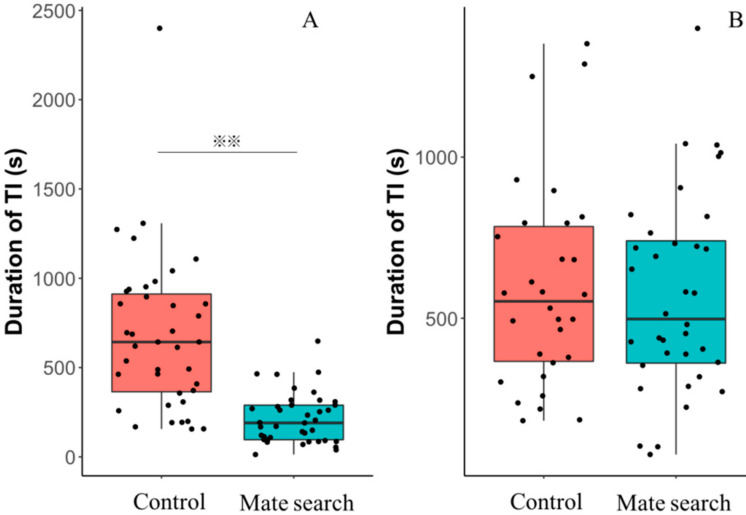
Comparison of the SPW from mate searching group (cyan) versus the control (orange) in duration of TI: (**A**) Duration of TI in male mate searching and control. (**B**) Duration of TI of female in mate searching and control. Box plots have a horizontal median, interquartile range box. Dots are experimental trial data points. Asterisks indicate a significant difference from the control (*p* < 0.01).

Within the males, the duration of TI of mate search group was significantly shorter than males in the control group (*X^2^* = 44.49, *p* < 0.01; Figure 3A). The duration of TI of males in the mate searching group was 214.53 ± 22.74 s (mean ± SE), and control group performed 679.64 ± 69.77 s. However, we did not observe a significant difference in the duration of TI between these two groups in the females (*X^2^* = 0.458, *p* = 0.50; Figure 3B). The mean duration of TI of females in the mate searching group was 569.64 ± 51.03 s, and control group performed 596.73 ± 58.08 s. At the same time, there were no significant differences between the duration of TI of males and females in the control group (*X^2^* = 0.383, *p* = 0.54; Figure 3).

### 3.3. Experiment 3: Effect on Mate Searching

**Figure 4:**
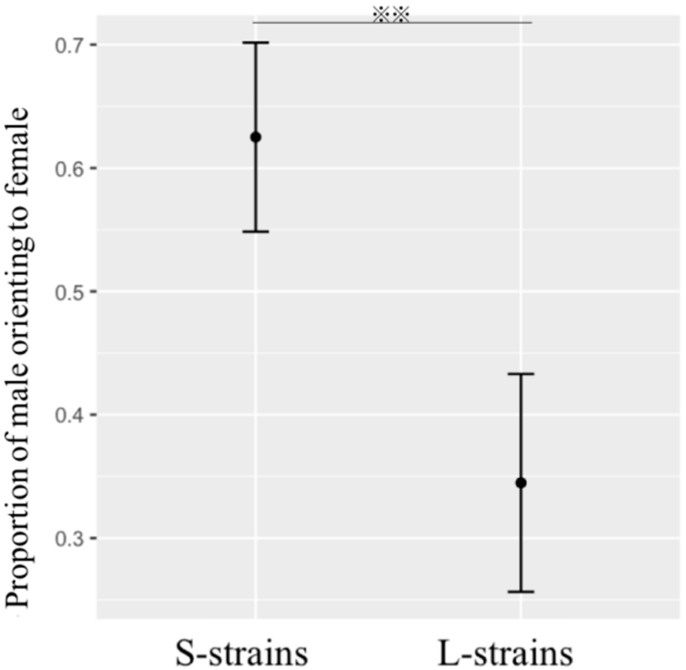
Differences between the SPW males from S-strain versus L-strain artificial regimes of their proportion of male oriented to females, error bars are 95% confidence intervals. Asterisks indicate a significant difference from the control (*p* < 0.01).

**Figure 5:**
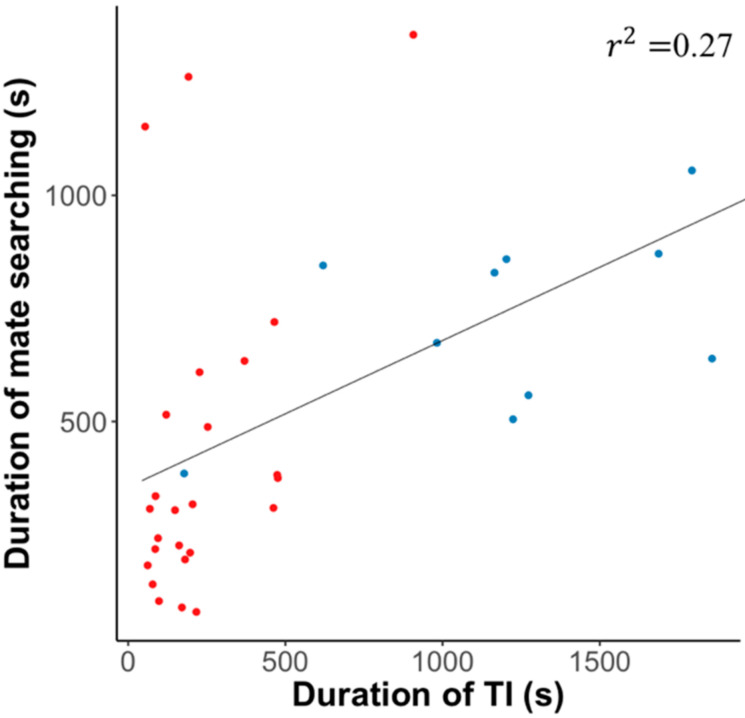
Scattergram for the correlation between the duration of TI and duration of mate searching (*y* = 345 + 0.329*x*) (*r^2^* = 0.27, df = 33, *p* < 0.01). Red dots are male SPW from S-strain. Blue dots are the male SPW from L-strain.

Before the experiment 3, we used Y-tube olfactometer as experiment 1 to measure the orientation of male in mate search each 6 h. Male from S (80% ± 8.9, *n* = 20) and L strain (60% ± 12.6, *n* = 20) both showed high probability of males being attracted to females during 21:00–2:59 h. The SPW males from the L-strain were 28% more likely to orient towards female attraction compared to the males from S-strain (*X^2^* = 5.352, *p* = 0.0207; Figure 4). 34.4% of L-strains males made response to females in the mate search experiment. In contrast, 62.5% of S-strains males made responses towards the females. Moreover, the correlation between the duration of TI (*T_TI_*) and mate search (*T_MS_*) of males which orientated to females is shown in Figure 5. The duration of mate search was significantly correlated with expression of TI (*r^2^* = 0.27, *p* < 0.01). We found the greater duration of TI, the longer duration of mate search in males of both artificial strains (S-strain *R^2^* = 0.22, df = 23, *p* < 0.05; L-strain *R^2^* = 0.25, df = 8, *p* > 0.05). The mean *T_TI_* and *T_MS_* of attracted males from S-srtain were 294.1 ± 61.4 s and 281.3 ± 54.6 s. The mean *T_TI_* and *T_MS_* of attracted males from L-srtain were 1310.2 ± 94.4 s and 722.0 ± 74.2 s.

## 4. Discussion

The results of this study confirmed that virgin males of the SPW prefers to search mate in the period of 21:00–02:59 h. This means that the most of male weevils start searching mate after 3 h of darkness. The TI was affected by mate search behavior in males because males showed a shorter duration of TI during the mate search. However, mate search did not affect the duration of TI in the females. Moreover, we determined that S-strain males of the SPW found their mates easier and faster than L-strain.

In the present study, we found more males orientated to females in the period of 21:00–02:59 h than other times (Figure 2). The period of 00:00–02:59 h was found to be the most active mate search time for the SPW under the controlled condition. So, male mate searching increased after 3 h at night. It may be due to the fact that females release more of their attraction pheromones at night than other times. As previous study reported, males of the SWP always mates with females from 16:00 to 04:00 h [40]. In this study, we further determined that the most active time of mate search at night. However, we still do not know the most active time of mate search of the SPW in the open field.

We hypothesized that males and females of the SPW invest in the mating behavior rather than in TI during the mate search. Our results indicated that males in mate search showed a significantly shorter duration of TI than males from the control (Figure 3A). The costs involved in the mate search are extensively debated. Possible costs involved in the expenditures and enhanced predation risks. The mate search is a costly behavior, resulting in less investment for anti-predator in both the males and females [32,48]. These results support our hypothesis in the males. The male keeps walking to look for females during mate searching. In previous research, we found that the duration of TI of the SPW is affected by the motion before the measurement. The SPW showed a decreased duration of TI when they are walking [6]. Meanwhile, previous studies found that the negative association between the mobility and TI [39]. The activity of all SPWs we measured were not mobile before the test. However, we did not placed males on gauze before experiment start. So, the male on the gauze must have moved during from the start to the end of the experiment.

However, no difference was found in the females tested (Figure 3B). Sometime, mate searching can be costly for females than the males [49]. However, in our study, there is no significant difference between the mate searching and control females. As the trade-off cost investment, the duration of TI of females would decrease during mate search. Because adult females of the SPW releases sex pheromone to attract males during the mate search [35]. However, female SPW usually attract males in a fixed position during mate search. As discussed above, mate searches did not alter the duration of TI in female SPW. It may also be caused by the provocation from the male weevils. Because male weevils will chase and mount female weevils during mate searching and courtship [50]. In order to prevent the intimidation from male, female invest in TI. The male weevils do not provoke female weevils under the TI.

As result in experiment 3, both strains showed high activity for the mate search at night. In experiment 3, we confirmed the hypothesis that the TI affects the mate search in the SPW. More males from S-strains oriented to females than L-strains mate search (Figure 4). That may be due to the higher levels of the candidate neuromodulator dopamine (DA) of the brain in S-strain. For example, in *Tribolium castaneum,* individuals from artificial S-strain (shorter duration of TI) have higher levels of brain DA than the *T. castaneum* from L-strain (longer duration of TI). At the same time, the S-strain beetle prefers to flee after an artificial threat [39]. Dopamine is an important factor in regulating insect activity and plays a key role in determining mate success [51,52]. Because the DA is considered to be related to spontaneous locomotor activity [53]. Moreover, some researchers found that male *Agrotis ipsilon* showed a lower proportion of orientating towards females in the group, when dopamine ecdysteroid receptor was sliced [54]. At the same time, the greater duration of TI, the longer duration of mate search was recorded in two strains (Figure 5). This means that with the investment of TI in two artificial strains, the ability of mate searching decrease.

We confirmed that L-strain showed longer duration of TI and shorter mate search activity. In the present study, we determined the effect of TI and mate search. So, we compared the duration of TI during the mate searching and resting in the SPW. At the same time the duration of mate was compared in two artificial selected groups. It helped us to understand the effect of mate searching on the TI. However, we did not measure the duration of TI of two artificial selected groups for the mate searching and resting.

We predicted that mate searching males and females invest in mate search behavior. In the present study, we were not able to find evidence that there is a trade-off investment between the mate search and TI behavior in females of the SPW. There are several possibilities for this outcome. First, mate searching behavior is not a costly behavior in female SPWs. Second, in order to prevent harassment from males, female SPWs invest in TI behavior. Third, we did not induce mate searching of female in experiment 2. It is unclear how to define whether a female weevil is searching for the mate. So, we found the most active mate searching period in a day in the present study. Up to 73% females attracted male weevils at midnight. The reason why females show no investment trade-off between TI and mate search behavior need further investigation.

## 5. Conclusions

Our study results provided a clear evidence that there is a trade-off investment between the mate search and TI behavior in males of the SPW. At the same time, we further know the most active time of mate search is at night. However, the investment trade-off in female needs further investigation. The trade-off between TI and mating behavior reflects the trade-off between anti-predator and reproduction. Insects need to find a suitable strategy based on the existing environment. In the present study, we highlighted the relationship between TI and mate searching behavior. Further, we quantified investment of anti-predator behavior and mating behavior by measuring the duration of TI and mate search in the SPW. Furthermore, we believe, these methods can be useful to determine the cost–benefit between anti-predator behaviors in the prey(s) and predator(s) in other species too.

## Figures and Tables

**Table 1:** The probability of males being attracted to females in eight different time periods.

Period	Probability of Orienting to Females (%)
00:00–02:59	73.3 ± 11.4 a
03:00–05:59	46.7 ± 12.9 b
06:00–08:59	40.0 ± 12.6 b
09:00–11:59	20.0 ± 10.3 b
12:00–14:59	26.7 ± 11.4 b
15:00–17:59	13.3 ± 8.8 b
18:00–20:59	26.7 ± 11.4 b
21:00–23:59	66.7 ± 12.1a

The letters indicate a significant difference in orientation rate of male (*p* < 0.05). Light condition (6:00–17:59) and dark condition (18:00–5:59).
